# Transcriptional Pathways Associated with Skeletal Muscle Changes after Spinal Cord Injury and Treadmill Locomotor Training

**DOI:** 10.1155/2015/387090

**Published:** 2015-08-24

**Authors:** Celine Baligand, Yi-Wen Chen, Fan Ye, Sachchida Nand Pandey, San-Huei Lai, Min Liu, Krista Vandenborne

**Affiliations:** ^1^Department of Physiology and Functional Genomics, University of Florida, Gainesville, FL 32610, USA; ^2^Center for Genetic Medicine Research, Children's National Health System, Northwest, Washington, DC 20010, USA; ^3^Department of Integrative Systems Biology, George Washington University, Northwest, Washington, DC 20010, USA; ^4^Department of Physical Therapy, University of Florida, Gainesville, FL 32610, USA

## Abstract

The genetic and molecular events associated with changes in muscle mass and function after SCI and after the implementation of candidate therapeutic approaches are still not completely known. The overall objective of this study was to identify key molecular pathways activated with muscle remodeling after SCI and locomotor training. We implemented treadmill training in a well-characterized rat model of moderate SCI and performed genome wide expression profiling on soleus muscles at multiple time points: 3, 8, and 14 days after SCI. We found that the activity of the protein ubiquitination and mitochondrial function related pathways was altered with SCI and corrected with treadmill training. The BMP pathway was differentially activated with early treadmill training as shown by Ingenuity Pathway Analysis. The expression of several muscle mass regulators was modulated by treadmill training, including *Fst*, *Jun*, *Bmpr2*, *Actr2b*, and *Smad3*. In addition, key players in fatty acids metabolism (*Lpl* and *Fabp3*) responded to both SCI induced inactivity and reloading with training. The decrease in *Smad3* and *Fst* early after the initiation of treadmill training was confirmed by RT-PCR. Our data suggest that TGF*β*/Smad3 signaling may be mainly involved in the decrease in muscle mass observed with SCI, while the BMP pathway was activated with treadmill training.

## 1. Introduction

Spinal cord injury (SCI) is one of the most disabling health problems faced by adults today. One of the physiological changes secondary to SCI is progressive muscle atrophy. The loss of muscle mass after spinal cord injury has been well documented, with patients with complete SCI having about 20–55% muscle atrophy [1] and patients with incomplete SCI showing about 20–30% atrophy [2] within 6 months to 1 year after SCI. Muscle wasting does not only impact the care and lifestyle of patients after SCI but also has significant health implications, increasing the patients' risk to develop secondary complications such as bone demineralization, diabetes, and cardiovascular disease [3, 4]. Maintenance of muscle mass is essential to metabolic health and is necessary to maximize functional gain from rehabilitation strategies after SCI.

A potential rehabilitation intervention in the treatment of individuals with SCI is the use of repetitive locomotor training to promote neural plasticity. This approach was derived from animal and human studies showing that stepping can be generated by virtue of the neuromuscular system's responsiveness to phasic peripheral sensory information associated with locomotion, in the presence of central pattern generator (reviewed in [5]). Our group and others have shown that locomotor training can induce substantial recovery in muscle size and muscle function in transected [6] and moderate contusion injury animal models of SCI [7–9]. Some studies suggest that this may reflect enhanced synthesis of growth factors within the central nervous system [10], but other potential activity-dependent molecular changes remain unknown, in particular the ones occurring locally in the muscle in parallel with atrophy and hypertrophy.

Muscle atrophy depends on the balance between protein breakdown, protein synthesis rates, and apoptosis. It has been attributed to the activation of various protein degradation pathways in several models of disuse, such as denervation, unloading, cachexia, or aging [11, 12]. Previous work in animal models showed that alterations in the protein ubiquitination and energy production related pathways are common features of the atrophy process [13–16]. The activation of growth factors such as insulin-like growth factors (IGFs), myogenic regulatory factors (MRFs) [17], transforming growth factors (TGFs) [18], and the bone morphogenic proteins (BMPs) [19] has also been shown to play an important role in muscle atrophy and hypertrophy. However, the activation or predominance of the different pathways involved can be specific to the condition inducing atrophy [20]. To our knowledge, only one study used gene profiling in human muscle samples to perform a general screening of the pathways activated in skeletal muscle after SCI [21]. However, the specific molecular signaling changes that occur after SCI and subsequent recovery and/or rehabilitation intervention remain largely unknown.

A better understanding of the molecular events regulating protein synthesis and degradation after SCI and locomotor training and their temporal relationship to changes in muscle mass is of considerable clinical importance and has far-reaching implications for posttraumatic health care. Therefore, the overall objective of this study was to identify key molecular pathways activated with muscle remodeling after SCI and during locomotor training. We implemented treadmill training in a well-characterized rat model of moderate SCI [9, 22–24] and performed genome wide expression profiling with microarray on soleus muscles at multiple time points during the course of SCI (prior and 3, 8, and 14 days after SCI) and during the course of a treadmill training intervention initiated 7 days after injury (8 and 14 days after SCI). We used two different approaches for data analyses. First, we took an unsupervised approach and identified molecular pathways affected most at each time point. We then targeted genes that are known to be involved in muscle remodeling and may play important roles in the process.

## 2. Material and Methods

### 2.1. Study Design

In this cross-sectional study, six groups of rats were studied, including a control group, three SCI groups at three time points (days 3, 8, and 14), and 2 SCI groups with treadmill training at two time points (days 8 and 14). In the latter groups, the treadmill was initiated 7 days after SCI. For the analysis of changes during the course of SCI, comparisons were done with reference to control samples for each time point after SCI (days 3, 8, and 14). For the analysis of the effects of treadmill training on SCI, samples from the treadmill trained SCI rats were compared to samples collected from SCI rats with no training. Differences between the trained muscles and untrained muscles at a specific time point (day 8 or day 14) were determined.

### 2.2. Animals

Thirty-six adult female Sprague Dawley rats (16 weeks of age, 260–280 g at the beginning of the study) were obtained from Charles River Laboratories and housed in a temperature (22 ± 1°C) and humidity (50 ± 10%) controlled room with 12:12 hours light:dark cycle. Animals were provided rodent chow and water ad libitum and were given 1 week to acclimatize to the environment. Animals were sacrificed at one of the following time points and conditions: 3 days after SCI (SCI3d, *n* = 6), 8 days after SCI (SCI8d, *n* = 6), 14 days after SCI (SCI14d, *n* = 6), 8 days after SCI and after 3 treadmill training sessions (SCI8d + TM, *n* = 6), and 14 days after SCI and after 5 days of repeated treadmill training sessions (SCI14d + TM). Age-matched control animals (CTR, *n* = 6) were used as the baseline. Experimental animals were given access to transgenic dough diet (Bio-Serv, NJ, product number S3472), placed on the bottom of the cage, to ensure adequate food intake. All procedures were performed in accordance with the US Government Principle for the Utilization and Care of Vertebrate Animals and were approved by the Institutional Animal Care and Use Committee at the University of Florida.

### 2.3. Spinal Cord Injury Procedure

All surgical procedures were performed under aseptic conditions. Moderate contusion SCI was produced using a NYU-MASCIS injury device as previously described [25, 26]. Briefly, the animals were deeply anesthetized with a combination of ketamine (90 mg/kg body weight) and xylazine (8 mg/kg body weight) and a dorsal laminectomy was performed at the thoracic vertebral level T7–T9 to expose the spinal cord [27]. Clamps attached to the spinous processes of T7 and T9 stabilized the vertebral column. Contusion was produced by dropping a 10 g cylinder from a height of 25 mm onto the T8 segment of the spinal cord. Analgesia was given in the form of buprinex (0.025 mg/kg) and ketoprofen (22 mg/kg) once daily over the first 36 hrs after SCI. The animals were housed individually and kept under vigilant postoperative care, including daily examination for signs of distress, weight loss, dehydration, and bladder dysfunction. Manual expression of bladders was performed 2-3 times daily, as required, and animals were monitored for the possibility of urinary tract infection.

### 2.4. Locomotor Training

Quadrupedal locomotor training was initiated on postoperative day 7. Training consisted of 20 min stepping sessions on a treadmill. Training was performed 3 times in the SCI8d + TM group (2 times on day 7 and one time on day 8) and was repeated twice a day for 5 days in the SCI14d − TM group. When necessary, body weight support was manually provided by the trainer. The level of body weight support was adjusted to make sure that the hindlimbs of the animals did not collapse and was gradually removed as locomotor capability improved. During the first day of training, assistance was provided to place the rat hindpaws in plantar-stepping position during training. Typically, rats started stepping when they experienced some load on their hindlimbs.

### 2.5. Tissue Collection

Left* soleus* muscles were harvested in all groups. In the SCI8d + TM and SCI14d + TM groups, muscle samples were harvested 8 hours after the end of the last treadmill training session. Briefly, rats were anesthetized with isoflurane (3% for induction, 1-2% for maintenance), and a small dorsal, midline incision was made to expose the gastrocnemius-soleus complex. The soleus was carefully separated from the gastrocnemius, harvested, and weighed. The sample was rapidly frozen in isopentane, precooled in liquid nitrogen, and subsequently stored at −80°C.

*t*-test was used to determine the statistical significance (*p* < 0.05) of the changes in muscle mass.

### 2.6. Expression Profiling

GeneChip Rat Genome 230 2.0 Array microarrays containing approximately 30,000 transcripts were used for the expression profiling experiment. Standard procedures including total RNA isolation, cDNA synthesis, cRNA labeling, microarray hybridization, and image acquisition were done as described in the manufacturer's protocol and our previous publications [20, 28]. Briefly, total RNA was isolated with TRIzol reagent (Invitrogen) and then purified with RNeasy MinElute Cleanup Kit (Qiagen). Two hundred nanograms of total RNA from each sample was reverse-transcribed to double-stranded cDNA followed by in vitro cRNA synthesis using one-cycle target labeling and control reagents and protocol (Affymetrix). Biotin-labeled cRNA was then purified using GeneChip Sample Cleanup Module (Affymetrix) and fragmented randomly prior to hybridizing to the microarrays overnight. Each array was washed and stained using the Affymetrix Fluidics Station 450 and then scanned using the GeneChip Scanner 3000. The quality control criteria developed at Children's National Medical Center Microarray Center for each array were followed [6].

Generation of hybridization signals of the microarrays was done using Microarray Suite 5.0 (MAS 5.0) (Affymetrix, CA) as previously described [21, 29–32]. After the absolute analysis, the gene expression values were imported into GeneSpring 11.0 (Silicon Genetics) for data filtering and statistical analysis. First, genes were filtered with numbers of present calls across the 36 arrays analyzed. Genes with at least 4 present calls (detected by more than 10% of the arrays) were selected for statistical analysis. We identified 31099 probe sets that met this filtering criterion. In GeneSpring, *t*-test was performed and probe sets showing significant (*p* < 0.05) expression changes were retained for pathway analysis. No additional fold change filters were used. The comparisons were done by comparing samples of each time point after SCI (days 3, 8, and 14) to the control time point (day 0), respectively. The treadmill trained samples were compared to the SCI samples collected at the same time point to obtain differences between the trained muscles and untrained muscles at a specific time point (day 8 and day 14).

To investigate molecular networks and pathways associated with gene lists in this study, Ingenuity Pathway Analysis (IPA) (Ingenuity Systems) was used with default settings to identify gene interactions and to prioritize molecular pathways differentially affected in different groups. Hierarchical clustering was performed using GeneSpring software to visualize transcripts showing coordinate regulation as a function of time.

The significance of the association between the genes in each dataset and the canonical pathway was determined by Fisher's exact test. The *p* values were calculated to determine the probability of the association between the genes. All profiles have been made publicly accessible via NCBI GEO (number GSE45550) (http://www.ncbi.nlm.nih.gov/geo/).

### 2.7. Reverse Transcription and Quantitative RT-PCR Analysis

Reverse transcription and quantitative RT-PCR (qRT-PCR) were performed as previously described [17]. Briefly, total RNA (2 *μ*g) was reverse-transcribed to cDNA using oligo(dT) primer (0.5 *μ*g/*μ*L) and reagents from Invitrogen. cDNA was amplified in triplicate in SYBR Green PCR Master Mix (Applied Biosystems). The thermal cycling conditions included 95°C for 10 min, followed by 40 cycles of amplification at 95°C for 15 s and 60°C for 1 min. Primer sequences used for rat myogenic factor 6 (MYF6 6/MRF4), follistatin (FST), mothers against decapentaplegic homolog 3 or SMAD family member 3 (SMAD3), and glyceraldehyde 3-phosphate dehydrogenase (GAPDH), which served as an internal control, are provided in Table 1. All primers were tested for nonspecific amplicons and primer dimers by visualizing PCR products on 2% agarose gels before performing qRT-PCR. The ΔΔCT value method (where CT is cycle threshold) was used to determine fold differences as described previously [17].

## 3. Results

### 3.1. Changes in Soleus Muscle Wet Weight after SCI and Treadmill Training

**Figure 1:**
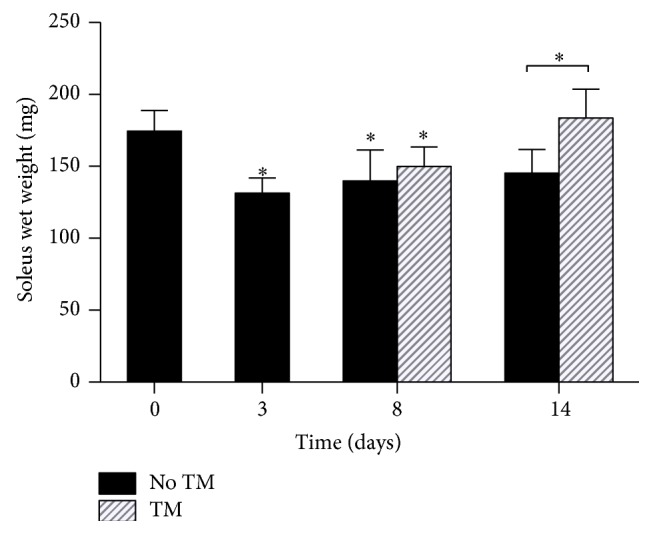
Muscle wet weight was significantly decreased on days 3 and 8 as compared to day 0 and significantly increased in the treadmill training group as compared to untrained animals on day 14. ^*∗*^ *p* < 0.05.

Soleus muscle wet weights are presented in Figure 1. SCI resulted in a rapid loss in muscle weight 3 and 8 days after injury (−25%, *p* = 0.0005). By day 14, muscle weight was still lower than in controls without reaching significance (−16%, *p* = 0.056). However, 5 days of training significantly increased muscle wet weight (SCI14d + TM, *p* = 0.005).

### 3.2. The Highest Number of Differentially Expressed Transcripts Was Observed 3 Days after SCI

**Figure 2:**
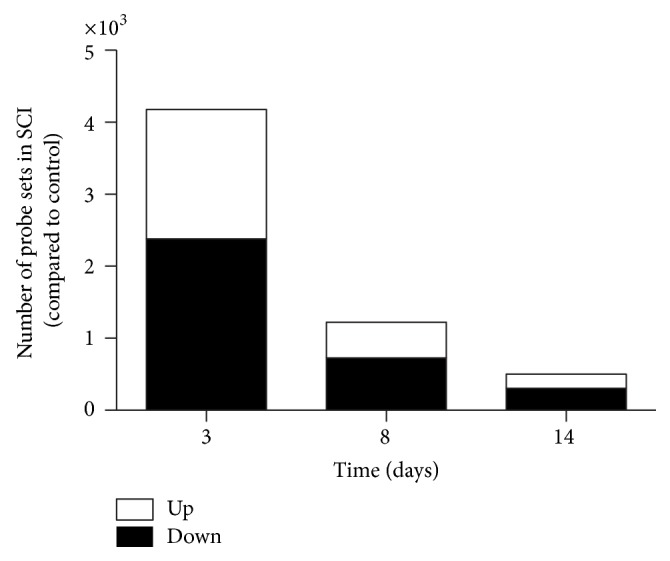
Number of probe sets differentially expressed in control and SCI rat soleus muscles decreased 8 and 14 days after SCI.

The largest number of genes differentially expressed compared to controls was found 3 days after SCI (Figure 2). In the treadmill trained groups, the largest changes were found on day 14, compared to untrained animals (Figure 2). In the untrained group, about 60% of the genes were downregulated at each time point, which was reversed in the treadmill training group on day 8 with 63% of the genes upregulated. To identify the major molecular pathways affected in each condition, Ingenuity Pathway Analysis (IPA) was performed.  Table 2 shows the top 5 canonical pathways for each comparison.

### 3.3. Protein Ubiquitination and ATP Production Related Genes Were Altered following SCI

At the early time point (day 3), we found that the protein ubiquitination pathway was largely activated. Indeed, 90% of the genes identified by IPA showed increase in gene expression. In particular, the expression of many proteasome subunits (PSMs) and ubiquitin specific peptidases and enzymes was increased (Suppl. Table  2 in Supplementary Material available online at http://dx.doi.org/10.1155/2015/387090). However, changes in this pathway were no longer significant on days 8 and 14 as compared to control levels. On the other hand, the mitochondrial dysfunction and oxidative phosphorylation pathways were consistently ranked among the top 5 most significant pathways at all time points of the study (days 3, 8, and 14) (Table 2). More than 95% of the genes identified in these two pathways were downregulated 3 days after SCI (Suppl. Tables  2, 3, and 4). In particular, the expression of the NADH dehydrogenase subunits (NDUF) genes, the succinate dehydrogenase complex subunits (SDH) genes, and the ATP synthase genes showed a decrease in gene expression.

### 3.4. Treadmill Training Rapidly Reversed the Changes in Protein Ubiquitination, Translation Factors, and Mitochondrial Function Pathway

The protein ubiquitination pathway was rapidly affected by treadmill training. While the majority of the PSMs were upregulated on day 3, an overall significant decrease was observed after 3 sessions of treadmill training. In the Eif2 and the eif4/p70sk6 pathways, the expression of the eukaryotic translation initiation factors (EIFs) subunits and kinases underwent overall negative fold changes on day 3 (Suppl. Table  2), which was corrected with training (Suppl. Table  4). In addition, the expression of NDUFs ATP synthases,* Cox8A* and* Cox7A2L*, was upregulated in trained animals on day 14, also showing a corrective effect of TM on muscle oxidative metabolism.

### 3.5. TGF-*β*/Smad and BMP Signaling Pathways Are Involved in the Muscle Remodeling Process during the Course of SCI and Early Treadmill Training

Because the bone morphogenic proteins (BMPs) signaling pathway was significantly altered on day 8 in the trained group (ranked number 3, *p* < 0.005), we examined the changes in gene expression of some key players in this pathway and in the TGF*β* pathway. To explore possible downstream changes, we also looked into the gene expression of the different smads associated with these pathways (*Smad1*,* Smad3*,* Smad4*,* Smad5*,* Smad6*, and* Smad8*), in particular on day 3, and with acute training on day 8.

On day 3 in the BMP pathway,* Smad4* (+1.8, *p* < 0.05),* Smad1* (+1.8, *p* < 0.05), and* Smad5* (+1.3, *p* < 0.05) gene expression was upregulated.* Smad3* did not show any significant changes on day 3, while the activin receptor 2B (*Acvr2B*) was downregulated (−1.6, *p* < 0.05). In parallel,* Smad6*, inhibitor of* Bmpr2* activation, was decreased (−2.3, *p* < 0.05).

**Figure 3:**
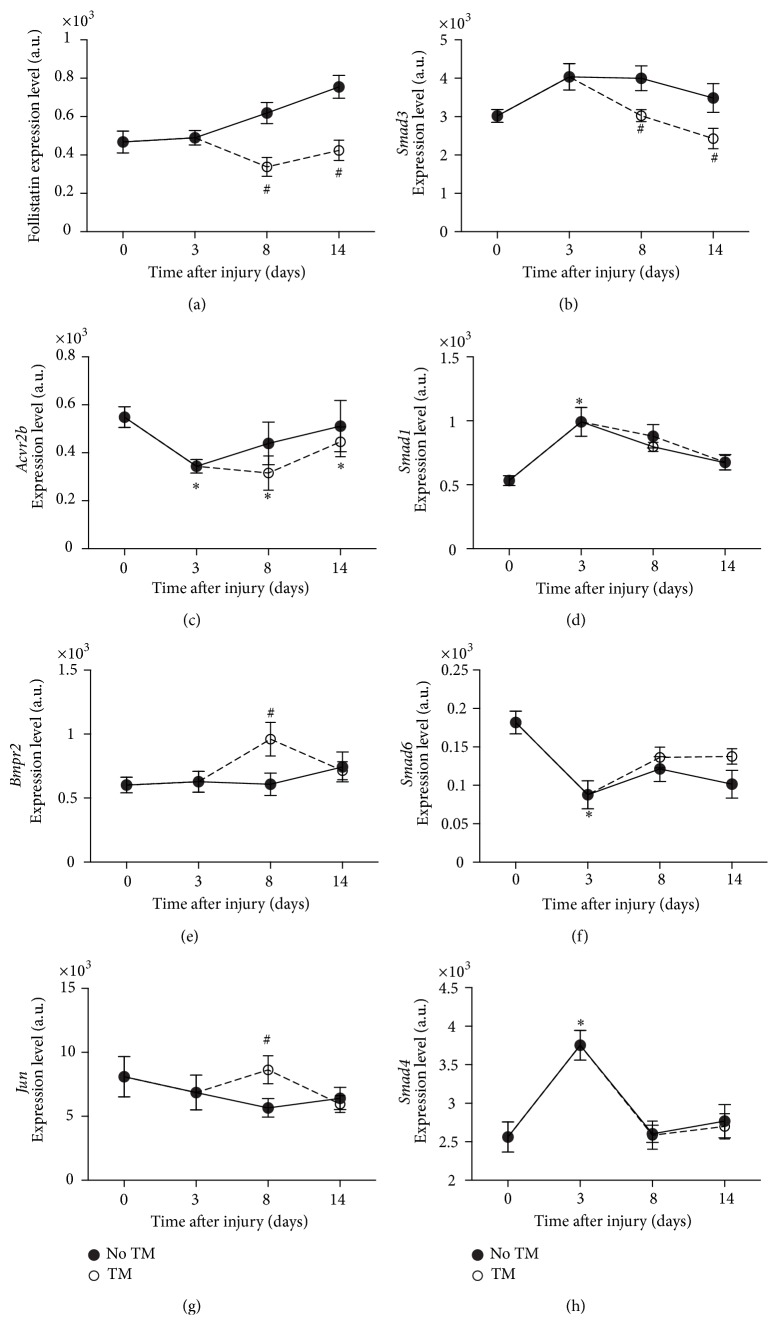
BMP/TGF*β* pathway related genes. Gene expression level changes in* Fst* (a),* Smad3* (b),* Acvr2b* (c),* Smad1* (d),* Bmpr2* (e),* Smad6* (f),* Jun* (g), and* Smad4* (h) in soleus of trained and untrained SCI animals. All expression levels are referenced to a control sample's GAPDH levels. ^*∗*^Significantly different from controls (*p* < 0.05). ^#^Significantly different from untrained animals (*p* < 0.05).

In SCI8d,* Smad1* gene expression was increased (+1.6 fold, *p* < 0.05). In addition, both follistatin (*Fst*) and* Smad3* expression were increased (+2.5 fold, *p* < 0.05, +2.0 fold, *p* < 0.05, resp.) as compared to controls (Figure 3).

In SCI + TM on day 8,* Fst* was significantly decreased (−1.5 fold, *p* < 0.05).* Smad3* was also found to be significantly downregulated (−1.3 fold, *p* < 0.05). The expression of the BMP complex 3 (*Bmp3*) was decreased (−2.35, *p* < 0.05). Importantly,* Bmpr2* was concomitantly overexpressed with treadmill training as compared to SCI only (+1.6 fold, *p* < 0.05). As part of the genes reported in the BMP pathway activated with training on day 8,* Jun* followed the same large and transient increase (+1.5, *p* < 0.05) as seen in* Bmpr2* (Figure 3).

### 3.6. Genes Involved in Myogenesis and Muscle Regeneration Were Affected by Treadmill Training

In addition to genes identified by IPA, we specifically studied genes involved in myogenesis, lipid metabolism, and fiber type switches, with a particular focus on those that were affected by 5-day treadmill training.

*Igfbp5*, modulator of IGF1 function in skeletal muscle, showed a significant decrease 3 days after SCI, followed by a large increase on day 14. This upregulation was not observed in the treadmill training group.

**Figure 4:**
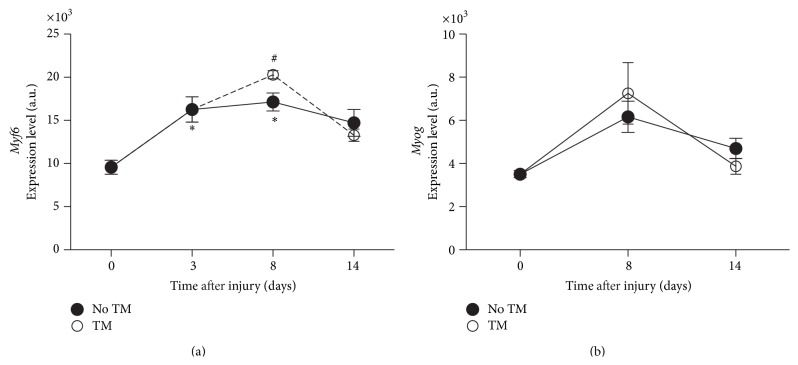
Myogenic regulatory factors. Gene expression level changes in* Myf6* (a),* Myog* (b), in soleus of trained and untrained SCI animals. All expression levels are referenced to a control sample's GAPDH levels. ^*∗*^Significantly different from controls (*p* < 0.05). ^#^Significantly different from untrained animals (*p* < 0.05).

Three days after injury, dramatic changes were observed in myogenic regulatory factors (MRFs), including* Myf6* and myogenin (*Myog*) (Figure 4). The* Myog* expression pattern was not affected by treadmill training. On the other hand, treadmill training induced an additional increase (*p* < 0.05) in* Myf6* after the first 3 sessions as compared to untrained animals.

### 3.7. Fatty Acid Metabolism and Fiber Type Switch Related Genes Showed High Sensitivity to SCI and Treadmill Training

**Figure 5:**
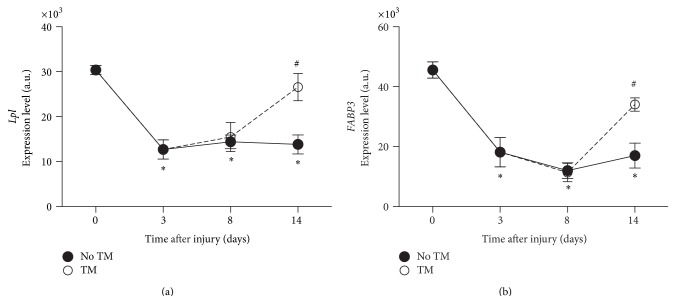
Fatty acids metabolism. Gene expression level changes in* Lpl* (a),* Fabp3* (b), in soleus of trained and untrained SCI animals. All expression levels are referenced to a control sample's GAPDH levels. ^*∗*^Significantly different from controls (*p* < 0.05). ^#^Significantly different from untrained animals (*p* < 0.05).

Lipoprotein lipase (*Lpl*) and* Fabp3* gene expression were dramatically decreased 3 days after SCI as compared to controls and maintained low expression levels on days 8 and 14 after SCI. While the first sessions of locomotor training did not significantly affect* Lpl* expression, it was completely restored on day 14 (+2.32 fold, *p* < 0.005) as shown in Figure 5.

**Figure 6:**
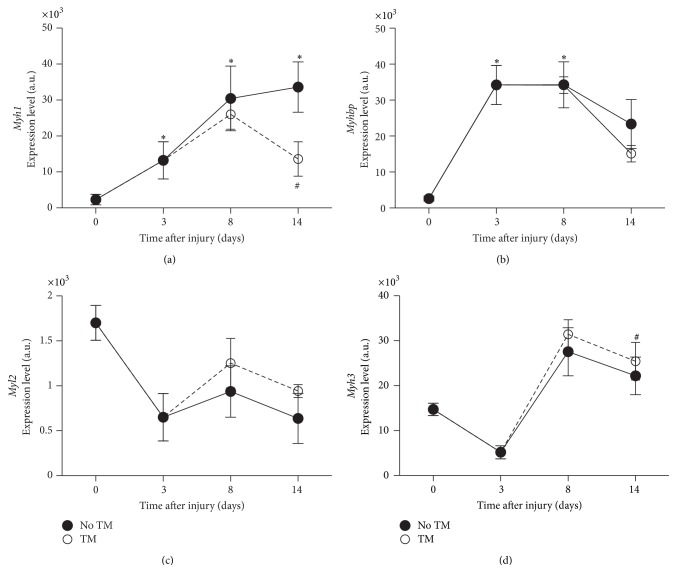
Myosin heavy and light chain indicative of fiber type. Gene expression level changes in* Myh1* (a),* Myhbp* (b), indicative of fast fibers, and* Myl2* and* Myh3*, indicative of slow fibers in soleus of trained and untrained SCI animals. All expression levels are referenced to a control sample's GAPDH levels. ^*∗*^Significantly different from controls (*p* < 0.05). ^#^Significantly different from untrained animals (*p* < 0.05).

As early as 3 days after SCI and throughout the experiment, several fast-twitch fiber markers genes (*Mhy1*,* Mybph*, and* Myh4*) showed large and significant changes (Figure 6). In parallel, the expression of several slow-twitch and vasculature smooth muscles markers (*Myh3* and* Myl2*) was decreased (*p* < 0.04) (Figure 6). Treadmill training had a significant reverse effect on the expression of some fiber type related genes, such as the fast-twitch markers* Myh1* and* Myh4*.

### 3.8. RT-qPCR Validation

**Figure 7:**
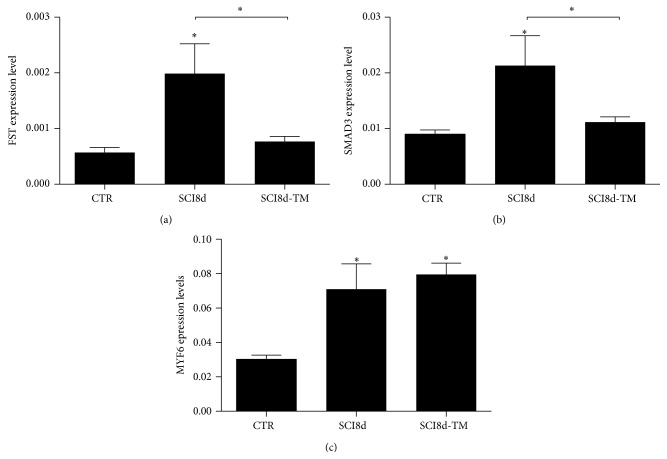
FST (a), SMAD3 (b), and MYF6 (c) mRNA expression in the soleus muscle of controls, untrained SCI animal on day 8 after surgery (SCI8d), and trained SCI animal on day 8 after surgery (SCI8d + TM). ^*∗*^ *p* < 0.05.

To validate microarray findings, we selected 3 genes (*Fst*,* Smad3*, and* Myf6)* that were found to be significantly affected by SCI and showed reversed changes after the initial treadmill training. RT-qPCR confirmed that* Fst* mRNA levels were significantly higher on day 8 after SCI compared to controls (+2.5 fold, *p* < 0.05) and decreased in the trained group compared to untrained (Figure 7).* Smad3* expression was also significantly decreased with acute treadmill training (−1.5 fold, *p* < 0.05). On the other hand, the increase in* Myf6* in SCI on day 8 was not confirmed (Figure 7(c)).

## 4. Discussion

The genetic and molecular events associated with changes in muscle mass and function after SCI and after the implementation of candidate therapeutic approaches are still not completely known. We used a well-characterized rat model of moderate SCI combined with treadmill training as a rehabilitation strategy to explore the pathways and genes involved in these conditions. The unique design of our study and the use of genome wide analysis allowed the identification of several major canonical pathways involved in protein synthesis and muscle metabolism regulation after SCI and treadmill training. In particular, the activity of the protein ubiquitination and mitochondrial function related pathways was altered with SCI and corrected with treadmill training. Of particular interest, the BMP pathway was differentially activated with early treadmill training as shown by IPA. The expression of several muscle mass regulators was modulated by treadmill training including* Fst*,* Jun*,* Bmpr2*,* Actr2b*, and* Smad3*. In addition, key players in fatty acids metabolism (*Lpl* and* Fabp3*) proved to be major sensors of SCI induced inactivity and reloading with training. The decrease in* Smad3* and* Fst* early after the initiation of treadmill training was confirmed by RT-PCR. Our data suggest that TGF-*β*/*Smad3* signaling may be mainly involved in the decrease in muscle mass observed with SCI, while the BMP pathway was activated with treadmill training. We also identified changes in fiber type markers, consistent with a switch towards type II fibers with SCI, and a reverse effect of treadmill training at the gene expression level as early as 1 day after initiation.

### 4.1. Acute Response from the Protein Ubiquitination Pathway to SCI and Training

The protein ubiquitination pathway is essential to the control of protein breakdown and turnover in the cell. It has been established that the activation of this pathway contributes largely to muscle wasting in multiple conditions [33], including denervation [34, 35], age and sarcopenia [36], and hindlimb suspension in rats [37] and in spinal cord injury in humans [21, 38]. In our model of moderate SCI, the protein ubiquitination pathway was activated as early as 3 days after injury, with the expression of 56 genes significantly upregulated. This upregulation was most likely responsible for the unbalance in protein synthesis/degradation ratio resulting in the large muscle wasting observed in the soleus on day 3. Conversely, the expression of genes in the protein ubiquitination pathway was very rapidly decreased on day 8 after only 3 sessions of treadmill training in SCI animals and the expression of several 20S proteasome subunits was significantly reduced. This was in accordance with previous observations in a hindlimb suspension rat model [39] 10 days after reloading. However, in another study in humans, Reich et al. [38] could not detect any reverse effect with 24 h of reloading after lower limb suspension. Here, we were able to observe a significant and early response within 36 h after the first training session, with a significant decrease in the expression of the genes related to protein ubiquitination (Suppl. Table  5).

### 4.2. Normalization of Mitochondrial Function Related Pathways with Training

Genes involved in the mitochondrial dysfunction and oxidative phosphorylation related pathways were also significantly changed. Together, the large number of NUDF, SDH, and ATP synthases with changing gene expression observed in SCI demonstrates a strong metabolic activity disturbance associated with SCI that does not completely recover within 14 days after surgery. Mitochondrial dysfunction after SCI was observed* in vivo* in several studies performed in humans with SCI [40, 41]. Our own work using ^31^P magnetic resonance spectroscopy to assess mitochondrial function* in vivo* in the same rat model of moderate SCI [24] showed that ATP production by mitochondria was affected 1 week after SCI and recovered in 3 weeks without training. It should be noted that, in the present study, no acute effect of treadmill training on mitochondrial dysfunction and oxidative phosphorylation pathways related genes was detected on day 8. However, our results suggest that treadmill training and the concomitant muscle hypertrophy are accompanied by partial correction of the mitochondrial function pathway genes (NUDFs and ATP synthase) at a later time (day 14), making mitochondria a target for early therapy in moderate SCI.

### 4.3. Role of TGF-*β*/Smad and BMP Pathways in Atrophy following SCI

**Figure 8:**
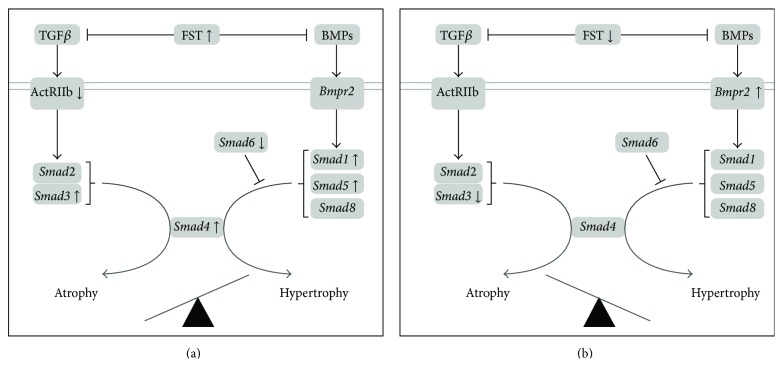
Schematic of the changes in soleus muscle gene expression within the TGF*β* and BMP pathways, with SCI (a) and acute response to treadmill training (b). Panel (a) shows the combined changes observed on day 3 and 8 after SCI, where several* Smads* (*Smads* 3, 4, and 1/5) and* Fst* gene expression was increased compared to control in parallel with muscle atrophy. Panel (b) summarizes the changes observed 8 days after SCI after 3 sessions of treadmill training compared to untrained animals. The changes in* Smad3* and* Fst* were reversed, and* Bmpr2* expression was increased, suggesting a role for the BMP pathway in the initiation of muscle mass recovery process observed with treadmill training.

Follistatin (FST) is well known as an inhibitor of the myostatin signaling within the TGF*β* pathway. It has been shown that* Fst* overexpression leads to a large increase in muscle mass in different animal models [42, 43]. Our results showed a large significant increase of FST mRNA levels with SCI after 8 days. It is possible that this increase reflects a protective mechanism intended to stimulate muscle growth. However our data showed that muscle wet weight did not further decrease after the initial 28% loss on day 3. One possibility is that the increase in FST mRNA levels may not have been sufficient to produce a recovery of muscle mass. Interestingly, we also observed a concomitant increase in* Smad3* and* Smad4* gene expression, downstream the Act2b receptor, which might be responsible for atrophy. Indeed, phosphorylated SMAD3 can mediate the activation of ubiquitin ligases that induces proteasomal degradation of contractile proteins [44]. More importantly, Winbanks et al. [45] recently showed that SMAD3 protein expression prevents skeletal muscle growth induced by follistatin and may suppress Akt/mTOR/S6K signaling. In addition,* Smad1* expression downstream the BMP pathway was increased, potentially competing with SMAD3 for the recruitment of SMAD4. If translated at the protein level, the balance between FST inhibition of the TGF*β* signaling and increased SMAD3 and SMAD1 expression may have led to a plateau in soleus muscle mass after the initial drop (Figure 8).

### 4.4. Role of TGF*β*/Smad and BMP Pathways in Hypertrophy following Treadmill Training

On the other hand, treadmill training was able to restore the initial muscle mass by day 14 after SCI. Our main associated findings were a large and rapid increase in the expression of* Bmpr2* with treadmill training 36 hours after initiation, in parallel with the significant decrease in FST and SMAD3 mRNA levels. The increased* Bmpr2* (BMPs receptor) levels could favor its activation by BMPs and the subsequent phosphorylation of Smad1, Smad5, and Smad8, which in turn bind with Smad4 leading to hypertrophy [19]. Whereas a decrease in FST expression has the ability to enable MSTN signaling and muscle growth, a parallel decrease in SMAD3 protein expression could contribute to an increase in SMAD4's availability to other binding proteins that can lead to hypertrophy, such as SMADs 1, 5, and 8. A concomitant and rapid increase in* Jun* was observed. It was shown that this transcription factor acts downstream the TGF*β* [46] and that overexpression of JUN results in dephosphorylation of SMAD3 [47]. This body of observations is suggestive of a deactivation of the TGF*β*/Smad pathway and a larger role for the BMP axis in the hypertrophy process following SCI and treadmill training.

### 4.5. Other Growth Factors (IGFs, MRFs)

The IGF1-Akt-mTOR pathway is known as a positive regulator of muscle mass [18, 48–50]. This pathway was not highly ranked by the present microarray analysis. However, the group previously demonstrated the impact of moderate SCI and treadmill training on several IGF proteins and binding proteins, but not on MYF5 in skeletal muscle [23]. Consistently with these previous results, here we observed that* Igbp5* and* Myog* were overexpressed after SCI and that* Igbp5* expression levels were corrected with treadmill training. In addition, we explored the possible impact of treadmill training on MRF4/MYF6 as indicated by the microarray results and found no significant increase in mRNA levels. This discrepancy may be due to experimental variations or slight biological differences between the batches of muscle samples used for microarray and RT-qPCR validation.

### 4.6. Fatty Acid Metabolism and Fiber Type Related Genes as a Sensor of Muscle Activity in SCI and Treadmill Training

Lipoprotein lipase (LPL) is a major enzyme involved in fatty acid (FA) metabolism and transport. After transport into the cytoplasm, FA binds to the fatty acid binding protein 3 (FABP3). Both LPL and FABP3 have been shown to be highly sensitive to contractile activity in muscle [51, 52]. Here, both* Lpl* and* Fabp3* genes showed high sensitivity to SCI induced disuse and to locomotor training. The downregulation of these fatty acid transporters in SCI rats was consistent with the recent observation by Long et al. [53] in muscle biopsies obtained from human subjects with SCI. On the other hand, we measured a large positive change in* Lpl* and* Fabp3* gene expression in response to locomotor training, with expression levels nearly corrected (92% of control levels) by day 14. Upregulation of LPL and FABP3 has been previously demonstrated in conjunction with increased muscle activity, such as muscle endurance training in humans [54], reloading after hindlimb suspension [28], and electrostimulation in denervated muscles [55] in rats. We established that treadmill training, as performed in our study, was sufficient to restore control levels of gene expression within 5 days. Our results further demonstrated the high sensitivity of* Lpl* and* Fabp3* gene expression to muscle activity and reloading achieved by treadmill training leading to muscle hypertrophy in moderate SCI rats.

Fiber type related genes also showed a great sensitivity to SCI induced disuse. It has been well established in the literature that muscle fibers distribution undergoes a shift towards fast fibers with disuse after SCI [56]. Consistent with this, we observed a fast change in the expression of myosin heavy and light chains genes, with an increase in some of the fast type (*Myh1*,* Myhbp*) and a decrease in the slow type (*Myl2* and* Myh3*). Interestingly, the expression level of* Myh1* was corrected in response to treadmill training on day 14, showing the effect of training on SCI muscles.

In summary, using genome wide analysis, we were able to identify some of the main pathways responsible for muscle wasting following moderate SCI and more importantly the corrective effect of treadmill training on these pathways. Here, we chose to focus on the BMP and TGF*β* signaling and established a key role for some of the genes within these pathways. Our observations suggest that* Smad3*,* Bmpr2*, and* Fst* are genes of interest in the study of moderate SCI. Protein expression and phosphorylation need to be investigated to allow further interpretation, although beyond the scope of this study. It would make it possible to test the hypotheses that* Fst* overexpression in SCI competes with* Smad3* regulation and that this effect is reversed by treadmill training leading to muscle mass recovery. More importantly, this would elucidate whether or not treadmill training activates the BMP/Smad pathway contributing to hypertrophy. This would establish the effect of BMP signaling activation and TGF*β* signaling on muscle regeneration with treadmill training in SCI via* Smad3* downregulation proving early indicators of efficient reloading in SCI rat and as a promising therapeutic approach. The body of data presented in this study constitutes a comprehensive guide to future studies targeting muscle mass preservation or recovery in moderate SCI.

## Supplementary Material

The supplementary material provides the following:1- A table of the top five canonical pathways affected at each comparison point and a list of the genes included in these pathways which expression levels were significantly changed.2- A series of five tables containing, each individual gene's accession number, expanded name, and fold change, for each of the top 5 pathways at each comparison point.

## Figures and Tables

**Table 1:** Primer sequences used for quantitative RT-PCR.

Genes	Primer	Primer sequence
*Gapdh *	Forward	F-5′-TCCGCCCCTTCCGCTGATG-3′
	Reverse	R-5′-CACGGAAGGCCATGCCAGTGA-3′

*Smad3 *	Forward	F-5′-AAGATACCCCCAGGCTGC-3′
	Reverse	R-5′-CTGTCTGTCTCCTGTACTC-3′

*Myf6 *	Forward	F-5′-CTAAGGAAGGAGGAGCAAG-3′
	Reverse	R-5′-TGTTCCAAATGCTGACTGAG-3′

*Fst *	Forward	F-5′-GTGTATCAAAGCAAAGTCTTG-3′
	Reverse	R-5′-GCTCATCGCAGAGAGCA-3′

**Table 2:** Five most significantly activated pathways for each comparison of the study.

Group/comparison	3 days	8 days	14 days
No treadmill training versus control	Protein ubiquitination pathway	Mitochondrial dysfunction	Mitochondrial dysfunction
	Mitochondrial dysfunction	Valine, leucine, and isoleucine degradation	Synthesis and degradation of ketone bodies
	Oxidative phosphorylation	Propanoate metabolism	Oxidative phosphorylation
	Regulation of eIF4 and p70S6K signaling	Butanoate metabolism	Citrate cycle
	Ubiquinone biosynthesis	Pyruvate metabolism	Ephrin receptor signaling

Treadmil training versus no training		Protein ubiquitination pathway	Estrogen receptor signaling
		Amyloid processing	EIF2 signaling
		BMP signaling pathway	Regulation of eIF4 and p70S6K signaling
		Role of BRCA1 in DNA damage response	Glutamate metabolism
		Hereditary breast cancer signaling	Granzyme B signaling
